# Current German practices in the prevention and management of postoperative pancreatic fistula following pancreatoduodenectomy: a nationwide survey

**DOI:** 10.1007/s00423-026-04012-7

**Published:** 2026-03-09

**Authors:** Irem Tacyildiz, Anke Mittelstädt, Christian Krautz, Georg F. Weber, Robert Grützmann, Maximilian Brunner

**Affiliations:** Department of General and Visceral Surgery, Friedrich-Alexander-University, Krankenhausstraße 12, 91054 Erlangen, Germany

**Keywords:** Pancreatic fistula, POPF, Partial pancreatoduodenectomy, Prophylaxis, Treatment, Survey

## Abstract

**Background:**

Postoperative pancreatic fistula (POPF) is the most frequent and clinically significant complication following pancreatoduodenectomy (PD) and represents the leading cause of postoperative mortality. Prevention, early recognition and adequate treatment are crucial for improving outcomes.

**Methods:**

A nationwide survey was conducted in October and November 2024, targeting 112 German hospitals routinely performing pancreatoduodenectomies. The questionnaire assessed surgical volumes, preferred anastomotic techniques, prophylactic and therapeutic strategies for POPF and postoperative monitoring practices. Data were collected via paper-based forms and QR code-assisted online formats.

**Results:**

A total of 77 hospitals, with an average annual volume of 45 PDs, participated in the survey (69% response rate). The pancreas was most commonly anastomosed with the jejunum (88%), with duct-to-mucosa pancreatojejunostomy (PJ) being the most frequently employed technique (39%). Abdominal drainage was routinely used in 91% of centers, with significantly higher rates observed in non-university and low-volume hospitals. Most participants estimated that 20–30% of cases involved high-risk anastomoses, primarily based on pancreatic texture and duct diameter. Only 18% of hospitals reported modifying their anastomotic technique in high-risk situations. As prophylactic measures, the use of somatostatin analogs (65%) and pancreatic duct stenting (36%) were the most common strategies, while prophylactic pancreatectomy was rarely performed (≤ 5% in 83% of centers). Therapeutic management of POPF primarily involved antibiotics (57%) and somatostatin analogs (54%), with interventional drainage – preferably CT-guided (82%) – required in 0–20% of cases, according to 96% of responses. Therapeutic pancreatectomy was reported to be mainly needed in 1–5% of cases. Neither prophylactic nor therapeutic management of POPF differed significantly by hospital type or volume.

**Conclusion:**

The survey reveals substantial variability in anastomotic techniques and in prophylactic and therapeutic strategies for POPF among German surgical centers, reflecting the current lack of high-level evidence in many areas. While minimally invasive management is widely adopted, the findings underscore the need for standardized protocols, improved risk stratification and further clinical trials to strengthen evidence-based practice in pancreatic surgery.

## Background

Postoperative pancreatic fistula (POPF) remains one of the most challenging and feared complications in pancreatic surgery, often referred to as the Achilles’ heel of the field. Despite advancements in surgical techniques and perioperative care, POPF continues to be the leading cause of postoperative morbidity and mortality following pancreatic resections, particularly after pancreatoduodenectomy (PD) [1–4]. Additionally, some studies have identified a prognostic impact of POPF on overall survival in patients with pancreatic cancer [5]. According to current literature, POPF occurs in approximately 20–30% of patients undergoing PD, with nearly half of these cases classified as clinically relevant (Grade B or C) based on the International Study Group on Pancreatic Surgery (ISGPS) criteria [1–4].

The occurrence of POPF is closely linked to the integrity and healing of the pancreatic anastomosis. Consequently, the choice of anastomotic technique plays a pivotal role in patient outcomes. The two most commonly employed reconstruction methods following PD are pancreatojejunostomy (PJ) and pancreatogastrostomy (PG). Various modifications of these techniques exist, including duct-to-mucosa and invagination methods, yet there is no consensus in the literature as to which approach is superior in minimizing the risk of POPF. Large randomized trials and meta-analyses have yielded conflicting results, further highlighting the lack of high-quality evidence guiding clinical decision-making in this context [6–10].

In addition to surgical technique, numerous patient- and procedure-related risk factors have been associated with POPF development. These include a soft pancreatic texture, small pancreatic duct diameter, high intraoperative blood loss, extended operative time and poor nutritional or general health status of the patient [3, 11–14].

As a result, the prevention, early detection and effective management of POPF are essential components of perioperative care in pancreatic surgery. A wide range of prophylactic and therapeutic strategies has been proposed, including the use of somatostatin analogs, pancreatic duct stenting, selective abdominal drainage and early imaging-based interventions [15–26]. However, practice patterns vary considerably between institutions and robust comparative data on their efficacy are still lacking.

In light of these uncertainties, a nationwide survey was conducted among German surgical centers to assess current clinical practices in the management of pancreatic anastomoses and POPF. The aim was to identify prevailing strategies, variation in approaches based on hospital type and volume and the perceived importance of risk stratification and technique adaptation in high-risk situations. The results of this survey may help to inform future research directions and support the development of standardized guidelines.

## Methods

### Study design and data collection

In October 2024, a total of 112 hospitals in Germany that regularly perform pancreatoduodenectomy (PD) were invited to participate in a nationwide survey. This cohort included all university hospitals as well as high-volume non-university centers, representing a broad cross-section of specialized pancreatic surgery institutions. In November 2024, a follow-up letter was sent to all non-responding hospitals. Data collection was primarily conducted using a paper-based questionnaire. To facilitate participation and improve response rates, a QR code linking to an identical online version of the questionnaire was also provided. The study was conducted in accordance with ethical standards. As the survey did not involve patient data, formal ethical approval was not required under local regulations.

### Questionnaire content

Our questionnaire encompassed the following key aspects:

- Annual volume of PD procedures.
- Number of surgeons performing PD at the institution.
- Standard technique used for pancreatic anastomosis.
- Routine use of abdominal drainage during PD.
- Criteria applied to define a high-risk pancreatic anastomosis.
- Estimated proportion of high-risk anastomoses.
- Modifications in anastomotic technique for high-risk cases.
- Prophylactic measures implemented in high-risk anastomoses.
- Frequency of intraoperative prophylactic pancreatectomy.
- Conservative measures used for the treatment of POPF.
- Estimated frequency and preferred technique for re-drainage placement.
- Frequency of therapeutic pancreatectomy due to POPF and whether concomitant splenectomy is routinely performed.
- Routine use of postoperative abdominal computed tomography (CT) prior to patient discharge.

### Definitions

High-risk pancreatic anastomoses were not predefined in the survey. Participants were asked to estimate the proportion of anastomoses they consider high-risk and to indicate the criteria they use. The routine use of validated risk scores was not specifically assessed, so classification reflects individual clinical judgment.

Completion pancreatectomy was defined according to the clinical context: prophylactic pancreatectomy refers to procedures performed intraoperatively as a preventive measure in cases of anticipated or evolving severe pancreatic fistula, excluding elective or oncologic indications, whereas therapeutic pancreatectomy refers to procedures performed after the occurrence of a clinically relevant postoperative pancreatic fistula (POPF) as a “rescue” strategy to manage severe complications, excluding elective or preventive indications.

### Statistical analysis

Data were analyzed using SPSS statistical software (Version 28; SPSS Inc., Chicago, IL, USA). Categorical variables were compared using the chi-square test, and continuous variables were analyzed with the Mann–Whitney U test. A p-value of < 0.05 was considered statistically significant.

Weighted mean prevalence values were calculated to summarize estimated frequencies across response categories. For each item, category midpoints were multiplied by their respective proportions, and the products were summed and divided by the total percentage (100%). This approach was used to provide an overall estimate of prevalence based on categorical survey responses.

## Results

### Participating hospitals

Of the 112 surgical hospitals in Germany contacted, 77 responded (69%), comprising 24 university hospitals (31%) and 53 non-university hospitals (69%). The median annual volume of pancreatoduodenectomies (PD) was 45 (range: 8–200), with university hospitals performing a significantly higher median number than non-university hospitals (75 vs. 35, *p* = 0.001). The median number of surgeons performing PD per hospital was 3 (range: 2–6) (Table 1).

**Table 1:** Characteristics of the participating hospitals

Response rate		77/112 (69%)
Hospitals	University hospitals Other hospitals	24/77 (31%) 53/77 (68%)
Median number of partial pancreatoduodenectomy per year [range]	All - University hospitals - Other hospitals	45 [8–200] 75 [8–200] 35 [15–80]
Median number of surgeons per hospital performing partial pancreatoduodenectomy [range]		3 [2–6]

### Surgical and postoperative standards of care regarding the pancreatic anastomosis

**Fig. 1:**
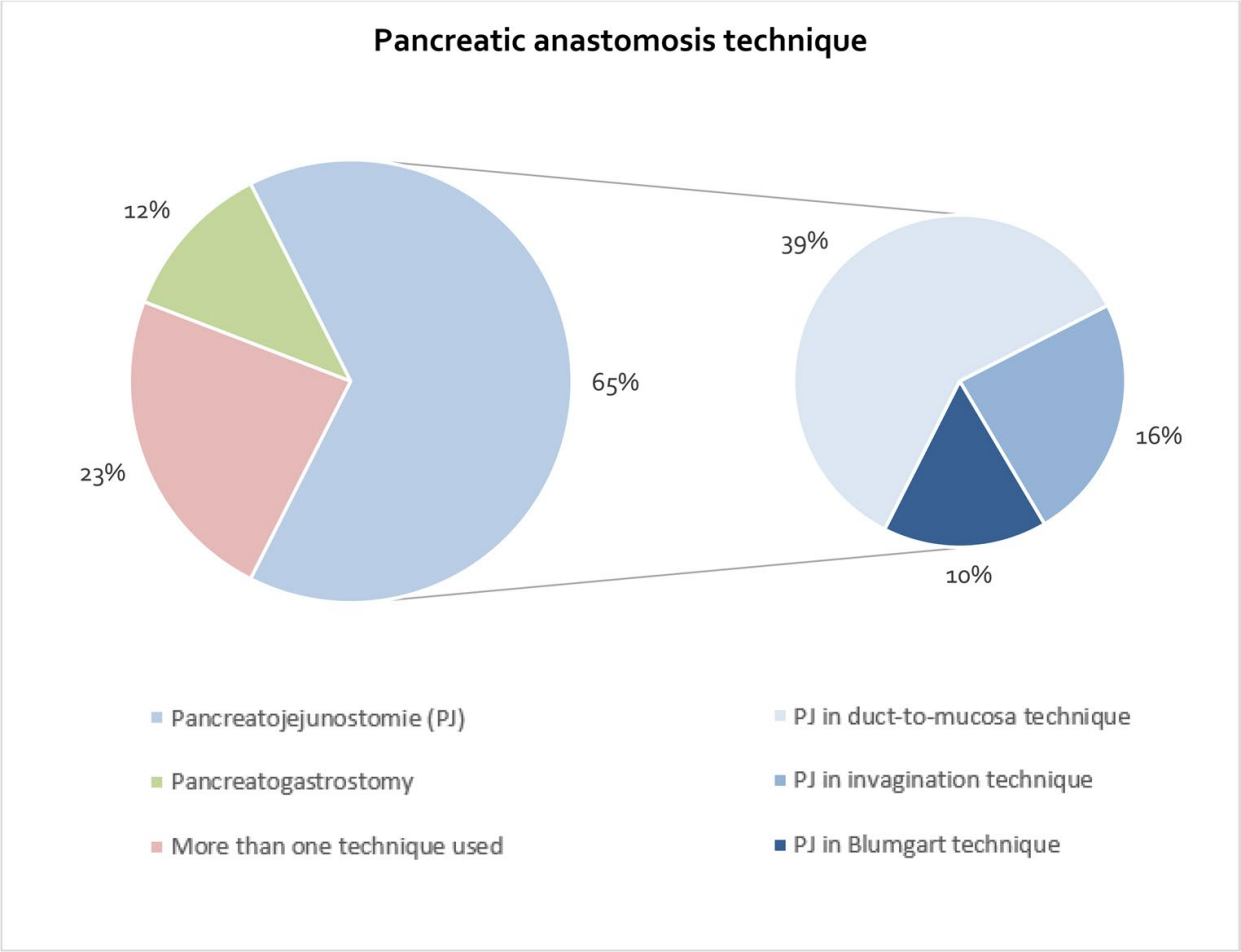
Technique used for pancreatic anastomosis among the survey participants (PJ = Pancreaticojejunostomy)

Among the responding hospitals, 59 (77%) reported adhering to a standard technique for pancreatic anastomosis, whereas 18 (23%) used more than one technique. The most commonly employed standard technique was pancreaticojejunostomy (PJ) with duct-to-mucosa anastomosis (39%), followed by PJ with the invagination technique (16%), pancreatogastrostomy (PG, 12%), and the Blumgart technique (10%) (Fig. 1). Preferences for these techniques did not differ significantly between university and non-university hospitals, nor did they vary with annual PD volume (Table 2).

**Table 2:** Standards for partial pancreatoduodenectomy

		All (*n* = 77)	University hospitals (*n* = 24)	Other hospitals (*n* = 53)	*p*-value	Hospitals with ≤ 45 PD/year (*n* = 41)	Hospitals with > 45 PD/Jahr (*n* = 36)	*p*-value
Technique used for pancreatic anastomosis, *n* (%)	PJ in duct-to-mucosa technique	30 (39)	11 (46)	19 (36)	0.623	13 (32)	17 (47)	0.464
	PJ in invagination technique	12 (16)	3 (13)	9 (17)		8 (20)	4 (11)	
	PJ in Blumgart techniqu	8 (10)	1 (4)	7 (13)		6 (15)	2 (6)	
	Pancreatogastrostomy	9 (12)	2 (8)	7 (13)		5 (12)	4 (11)	
	More than one technique used	18 (23)	7 (29)	11 (21)		9 (22)	9 (25)	
Use of drainage, n (%)	Always	70 (91)	19 (79)	51 (96)	**0.027**	41 (100)	29 (81)	**0.003**
	Depends on intraoperative situs	7 (9)	5 (21)	2 (4)		0 (0)	7 (19)	
Postoperative CT scan, n (%)	Always	2 (3)	0 (0)	2 (4)		1 (2)	1 (3)	1.000
	Only in case of signs of a complication	75 (97)	24 (100)	51 (96)	0.565	41 (98)	35 (97)	

*PD* Pancreatoduodenectomy, *PJ* Pancreaticojejunostomy; * Multiple answers possible

Abdominal drainage was routinely used in 91% of hospitals, while the remaining institutions used it selectively based on intraoperative findings. Non-university hospitals and hospitals with a lower annual PD volume (< 45 cases) more frequently employed drainage as standard practice compared to university hospitals (96% vs. 79%, *p* = 0.027; 100% vs. 81%, *p* = 0.003) (Table 2).

Only 2 hospitals (3%) reported routinely performing CT scans postoperatively in all PD cases, whereas 97% performed CT scans only when complications were suspected (Table 2).

### Prophylactic measures for preventing POPF in high-risk anastomosis

The estimated prevalence of high-risk anastomoses varied, with the highest proportion of hospitals (42%) reporting a frequency of 20–30%. University hospitals reported higher rates of high-risk anastomoses than non-university hospitals (*p* = 0.006). High-risk status was primarily assessed based on pancreatic texture (97%) and duct diameter (71%), with some also taking into account surgical intuition (31%) and the patient’s general condition (26%). Changes in anastomotic technique in high-risk cases were reported by 18% of hospitals, with no significant differences between hospital types or surgical volumes (Table 3).

**Table 3:** Prophylactic measures in high-risk anastomosis for the prevention of POPF

		All (*n* = 77)	University hospitals (*n* = 24)	Other hospitals (*n* = 53)	*p*-value	Hospitals with ≤ 45 PD/year (*n* = 41)	Hospitals with > 45 PD/Jahr (*n* = 36)	*p*-value
Estimated number of high-risk anastomoses, n (%)	≤ 20 %	24 (31)	4 (17)	20 (38)	**0.006**	15 (37)	9 (25)	0.157
	21 – 30 %	32 (42)	10 (42)	22 (42)		18 (44)	14 (39)	
	31 – 40 %	14 (18)	4 (17)	10 (19)		7 (17)	7 (19)	
	> 40 %	7 (9)	6 (25)	1 (2)		1 (2)	6 (17)	
Criteria for high-risk-anastomosis*, n (%)	Pancreatic texture Pancreatic duct diameter Surgical intuitionPatients` general condition	75 (97) 55 (71) 24 (31) 20 (26)	23 (96) 19 (79) 9 (38) 4 (17)	52 (98) 36 (68) 15 (28) 16 (30)	1.000 0.417 0.437 0.269	40 (98) 28 (68) 10 (24) 13 (32)	35 (97) 27 (75) 14 (39) 7 (19)	1.000 0.616 0.220 0.299
Change of anastomosis technique for high-risk anastomoses, n (%)	Yes No	14 (18) 63 (82)	3 (13) 21 (87)	11 (21) 42 (79)	0.298	9 (22) 32 (78)	5 (14) 31 (86)	0.269
Intra- and postoperative prophylactic measures for high-risk anastomoses*, n (%)	Somatostatin analogues Pancreatic duct stenting Antibiotics External drainage Dexamethason	50 (65) 28 (36) 11 (14) 10 (13) 6 (8)	15 (63) 11 (46) 3 (13) 2 (8) 4 (17)	35 (66) 17 (32) 8 (15) 8 (15) 2 (4)	0.800 0.309 1.000 0.493 0.072	29 (71) 14 (34) 8 (20) 4 (10) 1 (3)	21 (58) 14 (39) 3 (8) 6 (17) 5 (14)	0.339 0.813 0.203 0.501 0.092
Prophylactic pancreatectomy, n (%)	Never 1–5% 6–10% > 10%	31 (40) 33 (43) 10 (13) 3 (4)	9 (38) 10 (42) 4 (17) 1 (4)	22 (42) 23 (43) 6 (11) 2 (4)	0.947	15 (37) 18 (44) 6 (15) 2 (5)	16 (44) 15 (42) 4 (11) 1 (3)	0.923

*PD* Pancreatoduodenectomy; * Multiple answers possible

**Fig. 2:**
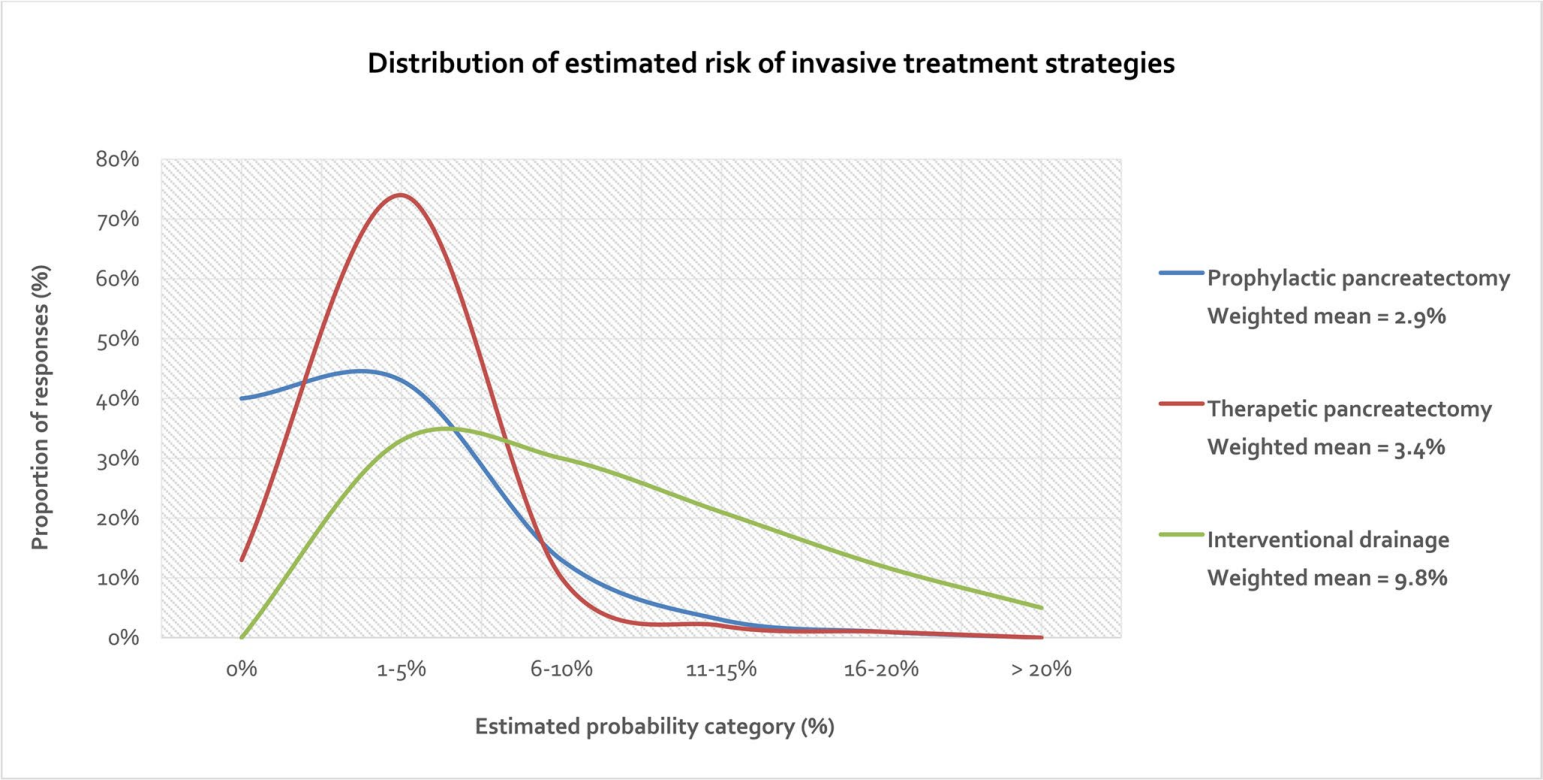
Distribution of estimated risk of invasive treatment strategies (prophylactic pancreatectomy, therapeutic pancreatectomy and interventional drainage) among the survey participants and their weighted mean risk

The most common prophylactic measures included somatostatin analogs (65%) and pancreatic duct stenting (36%), while antibiotics (14%), external drainage (13%) and dexamethasone (8%) were less frequently used. 60% of participating hospitals indicated that prophylactic pancreatectomy was performed in selected cases, with the majority of these (83%) reporting a prevalence of less than 5%. However, 17% of all hospitals reported performing prophylactic pancreatectomy in ≥ 5% of PD cases. The weighted mean prevalence was 2.9% (Fig. 2). There were no significant differences in prophylactic strategies based on hospital type or surgical volume (Table 3).

### Therapeutic measures for treating POPF

The most commonly used conservative treatments for POPF were antibiotics (57%) and somatostatin analogs (53%). The estimated need for additional interventional drainage was evenly distributed across the ranges < 5%, 5–10% and 10–20% (33%, 30% and 33%) with a weighted mean prevalence of 9.8% (Fig. 2). The most preferred drainage method was CT-guided external drainage (82%), followed by endosonographic internal drainage (22%) and sonographic-guided external drainage (7%). Therapeutic pancreatectomy for POPF was rare, with 47% of the hospitals reporting a prevalence of 1–2% and only 13% reporting a rate ≥ 5% resulting in a weighted mean prevalence of 3.4% (Fig. 2). During rescue pancreatectomy, splenectomy was the most commonly performed approach (32% always, 42% mostly). Therapeutic strategies for POPF showed no significant differences between hospital types or based on annual surgical volume (Table 4).

**Table 4:** Therapeutic measures for POPF

		All (*n* = 77)	University hospitals (*n* = 24)	Other hospitals (*n* = 53)	*p*-value	Hospitals with ≤ 45 PD/year (*n* = 41)	Hospitals with > 45 PD/Jahr (*n* = 36)	*p*-value
Conservative measurements for treatment of POPF*, n (%)	Antibiotics	44 (57)	14 (58)	27 (51)	0.626	25 (61)	16 (44)	0.174
	Somatostatin analoguess	41 (53)	16 (67)	28 (53)	0.323	26 (63)	18 (50)	0.258
	Flushing of existing drainage	9 (12)	2 (8)	7 (13)	0.712	2 (5)	7 (19)	0.074
Estimated number of need of postoperative drainage, n (%)	≤ 5%	25 (33)	7 (29)	18 (34)	0.942	15 (37)	10 (28)	0.764
	6–10%	23 (30)	7 (29)	16 (30)		10 (24)	13 (36)	
	11–20%	25 (33)	9 (38)	16 (30)		14 (34)	11 (31)	
	> 20%	4 (5)	1 (4)	3 (6)		2 (5)	2 (6)	
Preferred method of drainage*, n (%)	CT-guided external	63 (82)	21 (88)	42 (79)	0.529	32 (78)	31 (86)	0.393
	Endosonographic internal	17 (22)	5 (21)	12 (23)	1.000	7 (17)	10 (28)	0.284
	Sonographic-guided external	5 (7)	2 (8)	3 (6)	1.000	2 (5)	3 (8)	0.660
Performance of therapeutic pancreatectomy, n (%)	Never	10 (13)	5 (21)	5 (9)	0.588	6 (15)	4 (11)	0.361
	1–2%	36 (47)	10 (42)	26 (49)		18 (44)	18 (50)	
	3–5%	21 (27)	5 (21)	16 (30)		14 (34)	7 (19)	
	6–10%	8 (10)	3 (12)	5 (9)		2 (5)	6 (17)	
	> 10%	2 (3)	1 (4)	1 (2)		1 (2)	1 (3)	
Splenectomy during pancreatectomy, n (%)	Never	1 (1)	1 (4)	0 (0)	0.378	0 (0)	1 (3)	0.702
	Rarely	19 (25)	7 (29)	12 (23)		9 (22)	10 (28)	
	Mostly	32 (42)	10 (42)	22 (41)		17 (41)	15 (42)	
	Always	25 (32)	6 (25)	19 (36)		15 (37)	10 (28)	

## Discussion

This nationwide survey on surgical and postoperative practices in pancreatoduodenectomy (PD) across German hospitals achieved a relatively high response rate of 69% and therefore offers valuable insights into the current standards of care, particularly regarding pancreatic anastomosis techniques and the management of postoperative pancreatic fistula (POPF).

Regarding the pancreatic anastomotic technique, our findings demonstrate, on one hand, a considerable variability in the techniques used across hospitals. This aligns with current literature, which has not been able to establish the clear superiority of one anastomotic method over another [6–10]. On the other hand, there is also a notable degree of standardization within individual institutions: the majority of hospitals adhere to a single preferred technique, most commonly pancreaticojejunostomy (PJ) and specifically the duct-to-mucosa variant. Only about one-quarter of centers reported routinely using more than one anastomotic approach. This adherence is also reflected in the management of high-risk anastomoses, where only 18% of hospitals indicated they modify their technique. This practice pattern likely reflects the lack of evidence for a superior alternative and reinforces the continued use of a surgeon- or institution-preferred standardized technique. Surgeons rely on familiar techniques that have proven effective in their hands, ensuring technical precision rather than altering their approach in cases of high-risk anastomoses without clear evidence favoring one method over another.

Routine use of abdominal drainage was highly prevalent (91%) among the surveyed hospitals, with smaller and non-university centers employing this practice significantly more frequently. This trend may reflect a more conservative approach in settings with lower surgical volumes or more limited resources. However, a recent meta-analysis that included three randomized controlled trials (RCTs) found no significant benefit from the use of prophylactic drains and instead supports the omission of routine drainage in selected cases [15]. Despite this growing evidence, the adoption of modern drain policies - such as the omission of intraoperative drains or their early removal - has been remarkably slow among pancreatic surgeons. This discrepancy between evidence and practice is further reflected in a recent national survey from Italy, which similarly reported that the majority of surgeons continue to place prophylactic drains routinely after pancreatoduodenectomy [16].

Routine postoperative computed tomography (CT) after pancreatoduodenectomy (PD) was reported by only 3% of the surveyed hospitals, while the vast majority (97%) performed CT imaging only when clinically indicated. This selective use reflects a broadly adopted strategy of symptom-driven diagnostics, aiming to reduce unnecessary imaging and associated costs or risks. However, the role of routine CT in the early detection of postoperative complications remains a subject of debate [17]. Nevertheless, it is essential to note that the threshold for performing a CT scan should remain low in the presence of any clinical abnormalities, in order to enable timely diagnosis and management of potentially severe postoperative complications. This principle aligns with findings from the PORSCH trial, a multicenter stepped-wedge cluster randomized study, which demonstrated that the implementation of a structured, algorithm-based management strategy - including early diagnostics such as CT scanning when indicated - significantly improved postoperative outcomes in patients undergoing PD. The trial showed that standardized postoperative surveillance, incorporating early and consistent decision-making steps, reduced the rate of major complications and mortality [18].

The assessment and management of high-risk pancreatic anastomoses were also evaluated. 42% of hospitals reported a 20–30% prevalence of high-risk anastomoses, with pancreatic texture (97%) and duct diameter (71%) being the most commonly used criteria for risk prediction, which is consistent with the literature [3, 11–14].

Prophylactic strategies for preventing postoperative pancreatic fistula (POPF) in the context of high-risk anastomoses varied considerably across institutions. The most commonly applied measure was the use of somatostatin analogs, reported by 65% of hospitals. Despite this widespread use, the current literature presents conflicting results regarding their effectiveness [19, 20]. The continued use of somatostatin analogs, despite the uncertain evidence, is likely attributable to their ease of administration and favorable safety profile. The second most frequently reported prophylactic intervention was pancreatic duct stenting, employed by 36% of centers. However, the evidence supporting this strategy is also inconclusive [21]. The limited effectiveness and lack of standardization in technique may contribute to the relatively moderate adoption of this practice. In view of the substantial morbidity and mortality associated with POPF, some centers have considered prophylactic completion pancreatectomy in select high-risk cases. However, our survey data show that this is rarely used as a routine measure: only 13% of centers reported performing prophylactic pancreatectomy in more than 5% of PD cases. Most institutions indicated that this strategy was applied in only a small subset of cases (43% for 1–5%) or not at all (40%). These findings are consistent with recent meta-analytical data suggesting that prophylactic total pancreatectomy does not lead to a significant reduction in major morbidity or mortality [22]. Given the profound metabolic consequences of total pancreatectomy, this approach remains a highly selective and individualized measure, reserved for only the most extreme risk constellations [23, 24]. In summary, decisions appear to be driven largely by the invasiveness and simplicity of the measure as well as by the personal experience and clinical judgment of the surgeon and not by robust evidence.

The management of clinically relevant postoperative pancreatic fistula (CR-POPF) among participating centers demonstrated a clear preference for conservative treatment approaches. First-line therapies most commonly included antibiotics (57%) and somatostatin analogs (53%). In the case of proven infection, particularly abscess formation, the use of targeted antibiotic therapy - ideally guided by culture and sensitivity testing from intraoperative swabs or drain fluid - is a key component of effective management. However, the role of somatostatin analogs in the treatment of established CR-POPF remains controversial [25]. This conservative treatment strategy is broadly consistent with international guidelines, which recommend non-surgical management for lower-grade fistulas, particularly in clinically stable patients without systemic signs of infection or organ failure [26]. A notable proportion of hospitals reported the need for additional interventional drainage in up to 20% of CR-POPF cases. CT-guided percutaneous drainage was the most widely used technique (82%), followed by endoscopic ultrasound-guided internal drainage (22%) and ultrasound-guided percutaneous drainage (7%). These preferences reflect international best practices, wherein image-guided, minimally invasive drainage is considered the gold standard for managing symptomatic or infected fluid collections. It is generally regarded as superior to immediate surgical re-intervention in terms of patient outcomes and complication rates [27]. Surgical re-intervention, most commonly in the form of rescue pancreatectomy, was reported as a rare but necessary escalation in selected cases with refractory sepsis or progressive organ failure. While 47% of centers indicated a therapeutic pancreatectomy rate of 1–2%, only 13% reported applying this measure in more than 5% of cases. This underscores the high threshold for employing this radical strategy, which is associated with substantial morbidity due to the complete loss of both endocrine and exocrine pancreatic function. When performed, concurrent splenectomy was frequently necessary (32% always, 42% mostly), likely due to inflammatory changes or anatomical involvement complicating the procedure. In summary, the therapeutic management of POPF remains one of the most demanding challenges in pancreatic surgery. The data reflect a broadly accepted, stepwise, risk-adapted escalation approach that emphasizes minimally invasive and organ-preserving techniques. Surgical resection remains a last-resort option, reserved for severe, non-resolving cases where less invasive strategies have failed [26].

Importantly, no significant differences in therapeutic strategies were observed between university and non-university hospitals, or between centers with high and low procedural volumes. This suggests a general consensus across institutions regarding the stepwise escalation of POPF treatment, beginning with conservative measures and escalating to interventional or surgical approaches only when clinically necessary.

Several limitations of this survey must be acknowledged when interpreting the results. First, although the response rate of 69% is relatively high for a nationwide hospital-based survey, there remains a risk of selection bias. It is possible that centers with a particular interest in pancreatic surgery or those with more standardized practices were more likely to respond, potentially limiting the generalizability of the findings to all surgical institutions in Germany. Second, the study was designed as a descriptive survey aiming to provide a contemporary overview of reported clinical practice. It was not intended to explore causal relationships, determinants of practice variation or associations with clinical outcomes. Therefore, the findings should be interpreted as a structured national snapshot rather than as hypothesis-testing or outcome-driven evidence. As a consequence of this descriptive design, the study has limited novelty and conceptual depth, providing primarily a summary of current practice patterns rather than new mechanistic or outcome-based insights. Third, the data are based on self-reported practices derived from an anonymized nationwide survey rather than on audited institutional datasets or objective clinical outcome measures. As such, several responses necessarily reflect the respondents’ estimates rather than systematically documented figures. These estimates may be subject to recall bias and reporting bias and should therefore be interpreted with caution. In particular, key proportions represent subjective assessments rather than verified institutional data, which may further limit numerical precision. The results therefore reflect perceived practice patterns rather than validated outcome-based performance metrics, especially in areas subject to individual interpretation, such as the classification of high-risk anastomoses or the frequency of specific interventions. Fourth, the survey did not capture detailed patient-level data or stratify responses by specific clinical scenarios, which limits the ability to draw conclusions about the appropriateness or effectiveness of different management strategies in distinct patient subgroups. Finally, while the survey covered a broad range of relevant topics, it could not address every aspect of perioperative care. Important factors such as surgeon experience, team structure, postoperative monitoring protocols, and local infrastructure were not assessed, though they likely influence clinical decision-making.

## Conclusion

This survey highlights significant variability in the surgical and perioperative management of pancreatoduodenectomy across German centers. Despite heterogeneous evidence, most hospitals adhere to a standardized anastomotic technique and conservative strategies for POPF management. Practices often reflect clinical experience and institutional preferences rather than clear evidence, underscoring the need for further research and guideline development to improve standardization and outcomes in pancreatic surgery.

## Data Availability

No datasets were generated or analysed during the current study.
